# Dietary Fiber Regulation of Gut Microbiota and Bile Acid Metabolism in Animals: Implications for Animal Nutrition

**DOI:** 10.3390/vetsci13020209

**Published:** 2026-02-23

**Authors:** Jinhua Lai, Jürgen Zentek, Łukasz Marcin Grześkowiak

**Affiliations:** Institute of Animal Nutrition, Department of Veterinary Medicine, Freie Universität Berlin, 14195 Berlin, Germany; juergen.zentek@fu-berlin.de

**Keywords:** short-chain fatty acids, bile acid biotransformation, gut–liver axis, ruminants, non-ruminants, dietary regulation

## Abstract

Dietary fiber (DF) is a fundamental component of animal nutrition and has been widely studied for its nutritional and physiological functions in animals. While existing studies mainly focus on the independent effects of DF on gut microbiota or bile acids (BAs), the mechanisms underlying their interactions remain poorly understood. DF interacts closely with gut microbiota, promoting the production of beneficial metabolites such as short-chain fatty acids, which subsequently influence BA metabolism through microbial deconjugation and dehydroxylation processes, generating free and secondary BA essential for host health. Together, the gut microbiota and BA play key roles in mediating the effects of DF on intestinal and systemic physiology via the gut–liver axis. Although DF contributes to energy supply, nutrient digestion, and regulation of gut microbiota and BA metabolism, its physiological effects vary depending on fiber source, type, chemical composition, inclusion level, and animal species. Ruminant and non-ruminant animals differ in their capacity to utilize DF, with extensive fermentation occurring in the rumen of ruminants, whereas fermentation in non-ruminants mainly occurs in the hindgut and is more limited. Consequently, inappropriate DF supplementation may impair gastrointestinal function and overall physiological status. This review summarizes the diverse effects of different DF types in animals and critically examines the complex and bidirectional interactions among DF, gut microbiota, and BA metabolism, highlighting knowledge gaps that require further investigation to optimize DF application in animal nutrition.

## Simple Summary

Dietary fiber plays a crucial role in animal nutrition and gut health, and its digestion and metabolic pathways differ fundamentally between non-ruminant and ruminant animals. This review summarizes the interactions among dietary fiber, gut microbiota, and bile acids in both non-ruminant and ruminant animals, which vary depending on fiber type, source, and dosage. By highlighting these interactions, this review provides foundational information for livestock management, helping farmers and caretakers use dietary fiber more effectively. It also offers preliminary insights into the mechanisms of the dietary fiber–microbiota–bile acid axis, which can guide future research and improve animal health and productivity.

## 1. Introduction

Dietary fiber (DF) constitutes an important component of animal nutrition. Numerous studies have demonstrated that DF benefits gut health, metabolism, and immune function in livestock [1]. However, other studies have shown that excessive intake of DF may lead to digestive discomfort, diarrhea, and poor nutrient absorption, resulting in lower performance and even posing health issues [2,3]. Moreover, these phenomena manifest differently among various livestock species. At present, a diverse array of DF is incorporated into livestock diets, encompassing synthetic monosaccharide- or oligosaccharide-based compounds (e.g., polydextrose, fructooligosaccharides), purified plant-derived polysaccharides (e.g., cellulose, lignin, pectin), and structurally complex fiber matrices originating from whole plant sources such as vegetables, fruits, and cereal grains [4]. The complexity and diversity of DF in terms of its sources, types, composition, and physicochemical properties, combined with the presence of other nutrients (e.g., trace elements and vitamins) in natural DF sources, causes significant challenges in investigating the effects of DF on the gut health of livestock.

The animal gastrointestinal tract hosts trillions of microorganisms, including bacteria, archaea, fungi and viruses. These microorganisms establish complex, taxonomically diverse, and metabolically dynamic ecosystems collectively termed the microbiota [5]. The gut microbiota is essential for host development, nutrition, metabolism, physiology, and immunity. It supports these processes by metabolizing nutrients ingested by the host to produce transport proteins, enzymes, and receptor-interacting molecules [6]. The composition of gastrointestinal microbiota varies among different livestock species and is influenced by multiple factors, including animal genetics, location in the gastrointestinal tract, age, sex, diet, and environmental conditions [1]. DF is directly fermented by microorganisms in the gut, significantly impacting the composition of the gut microbiota [7]. Furthermore, the gut microbiota is recognized as a “metabolic organ”, deriving nutrients and energy from DF and producing various essential metabolites, such as short-chain fatty acids (SCFAs) or participating in biotransformation of BAs, which can regulate host metabolism by binding to homologous receptors and mediating related signaling pathways [8].

Bile acids (BAs) are a group of amphipathic steroid acids synthesized from cholesterol in all livestock animals [9]. After synthesis, primary BAs are transported to the intestine, where they are converted into secondary BAs by specific gut bacteria. As signaling molecules, BAs can bind to receptors and perform various physiological functions, including regulation of lipid, glucose, steroid, xenobiotic, and energy metabolism, as well as modulation of the host immune function [6]. There is a bidirectional interaction between BAs and the gut microbiota: the gut microbiota participates in the transformation and synthesis of BAs, while BAs influence the composition of the gut microbiota.

DF not only influences BA metabolism indirectly through microbial activity but can also act directly on BAs via binding and physicochemical interactions. Conversely, BAs can affect the composition of the microbiota, thereby influencing the metabolism of DF within the host. However, the crosstalk among DF, microbiota, and BAs is complex.

DF is a common feed ingredient in livestock diets and plays a critical role in animal health. Based on digestive physiology and feeding characteristics, animals can be categorized into non-ruminant animals and ruminants. Although gastrointestinal structure, digestive capacity, and BA composition differ among species, dietary DF act through similar metabolic pathways. This allows meaningful cross-species synthesis while acknowledging species-specific differences.

Therefore, the objective of this narrative review is to elucidate the effects of DF on different animal species within the framework of the DF–microbiota–BA metabolic axis.

## 2. Dietary Fiber

DF has a long history as part of carbohydrate fraction within food. But because of the complex structure and chemical compounds of DF, it is difficult to give a precise definition. The word “dietary fiber” was first proposed by Hipsley in 1953, who defined DF as the indigestible ingredient of plant cell wall [10]. However, the definition of DF has changed over many years [11,12,13]. In 2001, the American Association of Cereal Chemists (AACC) described DF as the edible part of plant or analogous carbohydrates that are resistant to digestion and absorption in the human small intestine with complete or partial fermentation in the large intestine [14]. This is a basic concept also useful in animal nutrition research. DF is generally considered to include polysaccharides, oligosaccharides, resistant starch, lignin, and associated plant substances [14,15].

### 2.1. Physicochemical Properties

DF is one of the plant-based feed sources for animals, providing energy for their growth and development while potentially imposing a digestive burden. The effect of DF depends on its source, type, physicochemical properties, and inclusion level, as well as the animal species and physiological status [16]. Some common DF sources and their composition added to livestock feed are listed in Table 1. Fiber fractions are presented according to established feed analysis systems, which describe soluble/insoluble fiber fractions and structural plant cell wall components and do not directly correspond to the physiological DF definition proposed by AACC.

DF is divided into different types and functions based on their origin, different locations in plant cell walls, chemical structures and components [4]. Moreover, this also determines that different DF have varied physical and chemical properties. The physicochemical characteristics of DF include particle size and bulk volume, surface area characteristics, hydration properties, adsorption or binding of ions and organic molecules, solubility, viscosity and fermentability [1,23].

DF can be classified based on its composition, oligosaccharide/polysaccharide type, physicochemical properties, and physiological role in digestion. However, these classification methods do not fully cover all fiber categories, and generally the most accepted classification of DF is based on its solubility and fermentability. The chemical structure of DF and its interaction with water molecules determine its solubility type. Based on solubility, DF is divided into two categories: soluble dietary fiber (SDF) and insoluble dietary fiber (IDF). The SDF mainly includes fructo-oligosaccharides (FOS), galacto-oligosaccharides (GOS), inulin, β-glucan, pectins, gums, psyllium, and others [24]. The insoluble fraction of DF includes cellulose, partial hemicellulose, and lignin, forming a linear and ordered crystal structure. The different solubility of DF has different effects on the digestion process of nutrients and microbial fermentation metabolism in the body. It has been reported that SDF is generally easily fermentable, whereas IDF is less fermentable by the gut microbiota [25]. In addition, the physicochemical characteristics affect the digestion of DF in animals and produce different physiological effects, for example, feed transit time in gut, fecal production and excretion, digestion and absorption of nutrients, bioavailability of trace elements, composition and metabolism of microorganisms and properties of BAs [11,26]. Lignin, a complex non-carbohydrate phenolic polymer, is particularly recalcitrant due to its highly branched and cross-linked aromatic structure. Unlike polysaccharide-based fibers, lignin is virtually non-fermentable in most non-ruminant animals and can physically encase other fiber components, reducing their digestibility and microbial accessibility [27].

### 2.2. Summary of Common Dietary Fiber and Its Components

Table 1 provides an overview of commonly used DF sources and ingredients in animal diets, highlighting their respective fiber fractions. SDF generally contribute to gut viscosity and fermentability, whereas IDF are associated with gut motility and fecal bulk. acid detergent fiber (ADF) and neutral detergent fiber (NDF) are measures of plant cell wall components: ADF reflects cellulose and lignin, while NDF includes hemicellulose in addition to ADF. Acid detergent lignin (ADL) indicates the lignin content, which can limit overall fiber fermentability.

The data illustrate considerable variation among fiber sources. For example, sugar beet pulp is notable for its high SDF content, making it a highly fermentable fiber source. In contrast, oat hulls and sorghum hulls exhibit high IDF and NDF levels, indicating a predominance of poorly fermentable structural carbohydrates. By contrast, cereal grains like corn, wheat, and barley contain lower overall fiber levels, with relatively low lignin contents.

### 2.3. Dietary Fiber Digestion and Fermentation Characteristics in Different Animals

The specificity of digestion of nutrients in different gut parts leads to spatial heterogeneity in the colonization of microorganisms in the intestine [18]. As one of the important sources of nutrients for animals, DF has different digestive characteristics in different animal species. 

In non-ruminant animals, DF largely escapes enzymatic digestion in the small intestine and undergoes microbial fermentation predominantly in the hindgut. However, certain fiber-degrading bacteria, such as *Ruminococcus*, *Butyrivibrio*, and *Prevotella*, colonize the stomach and proximal small intestine, partial digestion of DF occurs in the small intestine in pigs [28]. It should be noted that excessive or inappropriate DF supplementation may increase intestinal viscosity, reduce hindgut fermentation, decrease nutrient digestibility, and impair growth performance in pigs, with the magnitude of these effects depending on fiber source and production stage [29].

Adding SDF to the diet of chickens and other poultry can increase the intestinal viscosity, prolong the chyme passage through the gastrointestinal tract, create a hypoxic environment in the intestine, and reduce the growth of pathogenic bacteria [30]. In poultry, SDF can be fermented in the gizzard and duodenum, where the majority of the feed bolus is mixed with enzymes and mechanically ground in the gizzard [30]. Due to the lack of enzymes for degrading non-starch polysaccharides in poultry, the fermentation and utilization of IDF are limited [30]. Therefore, the crude fiber content in commercial diets is typically limited to 2–3%. However, adding a small amount (3–5%) of specific IDF such as cellulose to the poultry feed can improve gizzard function and nutrient utilization, but excessive supplementation may be counterproductive and act as an antinutritional factor, that affects the digestion and metabolism of nutrients by digestive enzymes [31]. 

In cats and dogs, most of the DF is fermented in the large intestine [32]. However, cats are carnivores with limited cecum and colon length, and certain DF such as cellulose and wheat bran are not extensively fermented [33,34]. Following microbial fermentation, DF serves as an energy source for microbes, supports microbial proliferation and influences chyme retention time in the gastrointestinal tract [32]. It can also contribute to the competitive exclusion of pathogens, stimulate the host’s production of antimicrobial compounds and modulate microbial colonization patterns in the intestines of cats and dogs [32]. However, inappropriate DF type or excessive inclusion, especially in feline diets, may reduce protein and fat digestibility and cause abnormal fecal characteristics or gastrointestinal discomfort [32,35].

Ruminant animals, in contrast to non-ruminants in which DF is mainly fermented in the hindgut after escaping small intestinal digestion, possess a specialized foregut fermentation system, where DF is extensively degraded before entering the lower gastrointestinal tract [36]. However, young ruminants have immature rumens at birth and rely mainly on the abomasum and small intestine for digestion, resulting in limited fiber fermentation. With increasing solid feed intake, rumen microbial communities gradually develop. Adult ruminants such as cattle and sheep have well-developed rumens, which contain a large number of DF-degrading microorganisms [36]. Roughage rich in DF is fermented by rumen microorganisms to produce organic acids and some primary fatty acids, which are absorbed delivering energy for the rumen epithelium and the intermediary metabolism [36]. The physical effectiveness and inclusion level of DF are crucial for maintaining rumen homeostasis. Insufficient fiber supply or imbalance in structural fiber proportion may impair rumination and salivary secretion, decrease ruminal pH, and increase the risk of ruminal metabolic disorders [37].

## 3. Dietary Fiber–Microbiota Interaction in Different Animals

Table 2 summarizes representative studies investigating how different DF sources modulate gut microbiota composition and, where reported, BA profiles across animal species. The table highlights variations in microbial responses to distinct DF types and provides an overview of associated changes in BA metabolism.

## 4. Microbial Regulation of Bile Acid Metabolism Across Animal Species

In the intestine, BAs are biotransformed by microbial-derived enzymes. BAs are synthesized by hepatocytes in the liver and stored in the gallbladder. After feed ingestion, they are released into the duodenum to emulsify fat. In the gut, intestinal bacteria metabolize and transform BAs through several processes such as deconjugation, dehydroxylation, oxidation, epimerization, and reconjugation, creating a diverse pool of BAs [6]. The diversity of the BA pool is shaped by the combined enzymatic activities of both the host and microbiota, with variations influenced by the composition of intestinal bacterial species, which differ across host species [92].

Microorganisms can influence gut health by regulating their metabolic products in the intestine, such as by secondary BAs. Studies have shown that secondary BA produced by the gut microbiota can stimulate the expression of P-glycoprotein in intestinal epithelial cells, thereby alleviating inflammation, maintaining colonic epithelial integrity, and mitigating colitis [93]. Additionally, research showed that a physiological level of two secondary BAs, such as deoxycholic acid and lithocholic acid, transformed by clostridia in the intestine, can inhibit the proliferation of harmful bacteria, such as *Clostridioides difficile* [94,95]. Furthermore, bacterial BA metabolism can promote the generation of peripheral regulatory T cells, contributing to immune regulation [96].

### 4.1. Types, Function and Physiological Role of Bile Acids

The composition of the BA pool differs among various livestock species. BAs can be detected in numerous biological fluids (such as bile, blood, urine, and follicular fluid), as well as in feces and liver tissue, with varying concentrations of primary BAs in different components. Based on origin, BAs are mainly classified into primary and secondary bile acids. Primary BAs are steroid molecules synthesized in the liver from cholesterol precursors such as cholic acid (CA) and chenodeoxycholic acid (CDCA) through various enzymatic reactions [97]. Secondary BAs are formed in the intestine from conjugated primary BAs through the action of the gut microbiota, undergoing 7α-dehydroxylation and 7α-dehydrogenation. The main secondary BAs include deoxycholic acid (DCA), lithocholic acid (LCA), ursodeoxycholic acid (UDCA), and hyodeoxycholic acid (HDCA) [98]. Primary and secondary BAs form their respective bile salts after conjugation with glycine or taurine. Conjugated BAs are more potent at fat emulsification, helping in fat digestion and energy provision to the host. The conjugated BAs mainly include: glycocholic acid (GCA), glycodeoxycholic acid (GDCA), taurocholic acid (TCA), taurodeoxycholic acid (TDCA), glycochenodeoxycholic acid (GCDCA), taurochenodeoxycholic acid (TCDCA), glycolithocholic acid (GLCA), and taurolithocholic acid (TLCA). Secondary BAs undergo further transformations, including epimerization, sulfation, glucuronidation, and conjugation with N-acetylglucosamine, resulting in a range of further modified bile acids, such as hyocholic acid (HCA), tauroursodeoxycholic acid (TUDCA), and sulfolithocholate (SLC). Additionally, BAs can be classified into two categories based on their structure: free BAs and conjugated BAs. Based on molecular groups, they can be further divided into hydrophilic BAs and hydrophobic BAs [9].

BAs are metabolic and immune signaling molecules that regulate various physiological processes in the body, such as glucose, lipid, steroid, and energy metabolism, by activating different BA receptors [6]. They also possess digestive-promoting, antioxidant, and antimicrobial properties, such as detoxifying endogenous or exogenous harmful compounds like bilirubin, bacterial lipid metabolites (endotoxins), and some inflammatory mediators [99].

### 4.2. Bile Acid Production and Metabolism

The synthesis of BAs is the primary pathway for cholesterol metabolism in animals. BAs are synthesized from cholesterol through oxidation catalyzed by cytochrome P450 (CYPs), followed by the synthesis of BA-CoA synthetase (BACS) and the conjugation catalyzed by BA-CoA: amino acid N-acyltransferase (BAAT) [6]. Primary BAs can be synthesized through four pathways: the classical pathway, the alternative pathway, the Yamasaki pathway, and the 25-hydroxylation pathway [100]. The classical and alternative pathways are the main routes for BA synthesis. In the classical pathway, cholesterol is hydroxylated by cholesterol 7α-hydroxylase (CYP7A1), which is the rate-limiting enzyme in the BA synthesis process. This is followed by conversion to CA through the actions of microsomal sterol 12α-hydroxylase (CYP8B1) and mitochondrial sterol 27-hydroxylase (CYP27A1). In the absence of CYP8B1, cholesterol is converted to CDCA. The alternative pathway begins with the activation of cholesterol by CYP27A1, converting it into 27α-hydroxycholesterol, and then 27α-hydroxycholesterol is hydroxylated by the rate-limiting enzyme 7α-hydroxylase (CYP7B1) to form CDCA [101].

The metabolism of BAs in the body is mediated via the enterohepatic circulation. In this process, hepatocytes synthesize BAs and secrete them into the bile canaliculi through active transport mechanisms involving key proteins such as the bile salt export pump (BSEP) and multidrug resistance-associated protein 2 (MRP2). The bile in the canaliculi then flows into the bile ducts surrounding the liver lobules and enters the gallbladder or directly into the duodenum. Following feed ingestion, cholecystokinin (CCK) is secreted by enteroendocrine I-cells in the duodenal and jejunal mucosa, stimulating gallbladder contraction and the release of bile into the small intestine to emulsify dietary fats. In the terminal ileum, approximately 95% of BAs are reabsorbed through the apical sodium-dependent BA transporter (ASBT) expressed on the apical membrane of enterocytes. BAs are co-transported with sodium ions through the ASBT into the enterocytes. Subsequently, BAs are transported across the basolateral membrane of enterocytes into the portal circulation via the organic solute transporters alpha and beta (OSTα/OSTβ). BAs are subsequently reabsorbed into hepatocytes through the sodium/taurocholate co-transporting polypeptide (NTCP) and organic anion-transporting polypeptides (OATP) located on the basolateral membrane of liver cells [92,101]. Additionally, other BA transporters in hepatocytes, such as MRP2 on the canalicular membrane and MRP3/MRP4 on the basolateral membrane, can transport BAs into the systemic circulation [102].

Multiple signals are involved in regulating BA metabolism, which affect intestinal immunity and health. Farnesoid X receptor (FXR) is a natural nuclear receptor for BA, highly expressed in the liver, intestine, and kidneys. FXR regulates BA metabolism by inhibiting the expression of rate-limiting enzymes in BA synthesis through three pathways. The first pathway involves BA activating FXR in the liver, which directly suppresses the expression of CYP7A1 [103]. The second pathway involves FXR inducing the expression of small heterodimer partner (SHP), which inactivates liver receptor homolog-1 (LRH-1), a signaling molecule necessary for the expression of CYP7A1, thereby inhibiting its expression [104]. The third pathway involves FXR promoting the binding of fibroblast growth factor 15/19 (FGF15/19) to fibroblast growth factor receptor 4 (FGFR4), which also inhibits the expression of CYP7A1. FGF19 is primarily expressed in the epithelial cells of the distal ileum, and the FXR-FGF19 pathway serves as the main BA sensing axis in intestinal epithelial cells [105].

Takeda G-protein-coupled receptor 5 (TGR5) is a BA receptor located on the cell surface, primarily expressed in the gallbladder and gastrointestinal tract. It plays an important role in regulating gallbladder relaxation and contraction, as well as in BA metabolism and intestinal function [106]. Upon binding with BAs on the surface of intestinal epithelial cells, TGR5 activates adenylate cyclase (AC), cyclic adenosine monophosphate (cAMP), and calcium signaling, leading to the release of glucagon-like peptides-1/2 (GLP-1/2) into the portal vein [107,108]. This represents another important BA sensing pathway in intestinal epithelial cells.

Other BA receptors in the intestine, such as pregnane X receptor (PXR), vitamin D receptor (VDR), and retinoic acid-related orphan receptor gamma t (RORγt), are mainly present in intestinal epithelial cells. Their expression and function are influenced by the intestinal microbiota [102,109]. VDR is a nuclear receptor that primarily regulates intestinal calcium absorption. Studies have shown that VDR plays a role in maintaining intestinal epithelial cell homeostasis. Increased expression of VDR in the mouse intestine can reduce epithelial cell apoptosis and maintain the intestinal mucosal barrier [110]. However, in mice with VDR knockout (KO) in intestinal epithelial cells, the expression of inflammatory factors, including tumor necrosis factor-alpha (TNF-α), monocyte chemoattractant protein-1 (MCP-1), and interleukin-1 beta (IL-1β) is elevated [111]. Moreover, *Carnobacterium maltaromaticum*, in combination with other microbes, can promote an increase in vitamin D levels and its metabolites in the colon of mice with colorectal cancer (CRC), as well as increase VDR activity in the mucosa, inhibiting CRC [109]. This suggests that VDR may play a potential role in maintaining epithelial cell homeostasis, preserving the intestinal mucosal barrier, and regulating immunity [112].

PXR is an important BA nuclear receptor that senses foreign compounds [113]. Research has shown that in mice receiving microbiota transplantation from ulcerative colitis patients, the expression of VDR and PXR in the colon is downregulated, enhancing inflammation [114]. Additionally, the microbial metabolite indole 3-propionic acid (IPA), as a ligand for PXR, can downregulate TNF-α expression in the intestine. When PXR is absent in the mouse intestine, intestinal barrier damage occurs, and the Toll-like receptor (TLR) signaling pathway is upregulated [115]. PXR is involved in regulating intestinal barrier function, downregulating inflammatory factor expression, and plays a key role in protecting the liver and intestine as well as regulating immunity.

RORγt is a BA nuclear receptor associated with multiple inflammation and immunity-related processes [116]. Studies have found that BA, after being metabolized by the microbiota, can regulate the expression of the transcription factor RORγt in an important population of colonic Foxp3+ regulatory T cells (Tregs), thereby enhancing mucosal immune tolerance and reducing susceptibility to inflammatory bowel disease—an effect mediated in part through other BA receptors such as the vitamin D receptor (VDR) [117]. Further studies have shown that innate lymphoid cells type 3 (ILC3) promote microbiota-specific RORγt+ Tregs and prevent their expansion into inflammatory Th17 cells, thereby defending against aberrant immune responses and inflammation in the microbiota and establishing intestinal tolerance [118].

### 4.3. Microbial Metabolites Regulate Bile Acid Metabolism and Immunity via Bile Acid-Related Signaling Molecules

SCFAs, as the main products of DF microbial fermentation in the intestine, participate in BA metabolism and immune regulation by modulating multiple BA-related signaling pathways. From the perspective of bile acid metabolism, SCFAs suppress BA synthesis by inhibiting Cyp7a1 through the intestinal FXR-FGF15 axis, thereby improving BA homeostasis [119]. Acetate and propionate activate the G protein-coupled receptors FFAR2 (GPR43) and FFAR3 (GPR41) on intestinal epithelial and enteroendocrine cells, promoting GLP-1 secretion and indirectly regulating bile acid metabolism by modulating intestinal function, energy homeostasis, and bile secretion [120]. Butyrate inhibits histone deacetylase (HDAC) activity and enhances histone acetylation, which increases PXR transcription and promotes PXR-mediated expression of the glucose transporter GLUT2 and P-glycoprotein (ABCB1), accelerating cholesterol metabolism and transport [121]. From an immunological perspective, butyrate activates intestinal VDR expression and related signaling pathways, enhances intestinal epithelial barrier integrity, and exerts anti-inflammatory effects [122]. In addition, butyrate suppresses Th17 cell development by inhibiting RORγt expression and increases IL-10 production in T cells, which may be involved in colitis development [123].

### 4.4. Microbes Influence Bile Acid Metabolism in the Intestine of Different Animals

Table 3 summarizes representative studies demonstrating the direct involvement of gut microorganisms in BA metabolism across different animal species. The table highlights the role of microbial interventions in regulating BA transformations, including deconjugation, dehydroxylation, and conversion between primary and secondary BA. These findings indicate that the gut microbiota plays a central role in shaping BA pools through enzymatic and metabolic activities.

Free primary BAs in the liver are further modified by the gut microbiota during the BA enterohepatic circulation, and there are various physiological processes of mutual influence between gut bacteria and BAs. However, these physiological processes primarily depend on the expression of functional bacterial proteins related to BA metabolism, including enzymes associated with BA microbial transformation and BA transport proteins [135]. The gut microbiota can promote the secretion of intestinal enzymes, thereby influencing BA synthesis [136]. BSH is mainly found in genera such as *Clostridium*, *Bacteroides*, *Bifidobacterium*, *Lactobacillus*, *Enterococcus*, and *Listeria*. BSH dissociates and binds BAs by hydrolyzing the amide bond between the glycine and taurine parts that are attached to the steroid core of bile salts, which is the critical first step in microbial transformation of BAs [102,103,137,138]. 7α/β-dehydroxylases are mainly found in anaerobic microorganisms in the gut, such as *Clostridium* and *Eubacterium*, where they catalyze the 7α- or 7β-dehydroxylation of glycine- or taurine-conjugated BAs like CA and CDCA, converting them into secondary BAs such as DCA and LCA [102]. 3α-/3β-/7α,β-/12α,β-hydroxy steroid dehydrogenases (HSDHs) have been detected in *Clostridium* species, and these enzymes play a crucial role in BA metabolism by oxidizing or reducing hydroxyl groups at various positions on the BA molecule, thereby altering the structure and activity of BAs [102]. Furthermore, *Clostridium* species also express the proton-dependent BA transporter BaiG, which mediates the entry of free primary BAs into the bacteria [139]. Additionally, microbes can influence BA metabolism by mediating related BA receptor signaling [140].

As shown in Table 3, in non-ruminant animals, microbial interventions, including fecal microbiota transplantation and targeted probiotic supplementation, have been shown to alter bile acid composition and modulate microbial enzymatic activities related to bile acid metabolism in pigs and poultry. Studies have demonstrated that supplementing piglet diets with *Bacillus subtilis* fermented liquid enhanced the growth of bile salt hydrolase-active and 7α-dehydroxylase-active bacteria in the intestine, disrupting the normal production of unconjugated BAs [126]. In dogs and cats, *Peptacetobacter* (*Clostridium*) *hiranonis* and certain members of Oscillospirales have been confirmed as key bacterial species responsible for converting primary BAs into secondary BAs. These bacteria contain functional bai operons and influence the composition and function of BA through specific enzymes such as BSH [130,131].

In ruminants, rumen microorganisms and microbial additives participate in bile acid metabolism through diverse enzymatic pathways and microbial enrichment.

### 4.5. Bile Acids Influence the Composition of Microbiota in Different Animals

Table 4 summarizes representative studies investigating how BA interventions, including exogenous BAs, BA extracts, and BA receptor agonists, influence gut microbiota and host physiological responses in different animal species. The table highlights BA-mediated antimicrobial effects, receptor signaling pathways, and shifts in microbial community structure associated with altered BA pools. These findings reveal the bidirectional interaction between bile acids and the gut microbiota within the DF–microbiota–BA axis.

BAs influence gut microbial composition through multiple interconnected mechanisms. BAs exert direct antimicrobial effects by disrupting bacterial membranes and inhibiting the growth of bile-sensitive taxa, thereby selectively shaping microbial communities [8]. BAs can modulate the intestinal environment and nutrient availability, indirectly affecting the competitive fitness of different microbial populations [8]. In addition, BA signaling via host receptors, such as FXR and TGR5, regulates intestinal immune responses and barrier function, creating ecological niches that favor specific bacterial taxa [8,154]. The effects of BAs on gut microbiota are highly dependent on BA species, dosage, dietary context, and host physiology. Together, these mechanisms contribute to species-specific and diet-dependent remodeling of gut microbial communities.

As shown in Table 4, in non-ruminant animals, supplementation with specific bile acids or bile acid-related compounds generally modulated the abundance of dominant bacterial taxa, including *Prevotella*, *Lactobacillus*, and *Bifidobacterium*, and affected overall microbial diversity. However, a study also reported no significant effects of dietary bile acid supplementation on gut microbiota in pigs [144], which may be related to differences in bile acid type and dosage. In poultry, bile acid supplementation under different dietary conditions produced distinct effects. For example, under low-fat diets, supplementation with HCA, HDCA, and CDCA increased the abundance of *Akkermansia* in the cecum, whereas under high-fat diets, bile acid supplementation reduced the relative abundance of *Bacteroides* and increased *Bifidobacterium*, *Escherichia coli*, and *Lactobacillus* [149]. In broilers, bile acid supplementation was frequently associated with reduced colonization of pathogenic bacteria, such as *Clostridium perfringens* and *Campylobacter jejuni*, and enrichment of beneficial genera [146,147,148].

In ruminants, BA supplementation altered the relative abundance of BA- and DF-responsive bacteria, including decreased *Prevotella* and *Treponema* and increased *Akkermansia*. In addition, microbiota-derived BA, such as ursodeoxycholic acid, have been shown to inhibit antibiotic-resistant bacteria, including ESBL-producing *Escherichia coli*, in dairy calves [100].

## 5. The Mechanisms by Which Dietary Fiber Affects Bile Acid Metabolism

DF affects BA metabolism in livestock animals through various mechanisms. DF has the ability to adsorb BAs, influencing their reabsorption in the animal body and increasing the excretion of BAs in feces [155]. Additionally, DF can alter the passage rate, thus influencing the excretion of BAs from the gut. Furthermore, DF can modify the microbial composition in the animal intestine, affecting the enzymatic activity of bacteria involved in BA conversion, thereby influencing the microbial transformation of BAs [156,157].

### 5.1. Binding of Dietary Fiber and Bile Acids

DF can directly influence the structure and composition of BAs by binding to them. The binding of DF to primary BAs is an effective method to reduce cholesterol levels in the blood, while binding to secondary BAs can reduce the toxic effects of these metabolites on colon cells. Studies have shown that different bile salts have varying binding affinities for pectin, with the order being GCA > GDCA > CA > DCA > CDCA > GCDCA. As for cellulose, its binding affinity to bile salts is in the order of GDCA > CA > GCA > DCA > CDCA > GCDCA [100].

After mixing citrus pectin with BA, an endothermic reaction occurs, and the concentration of BA decreased. This result suggests that there is some interaction between BA and pectin [158]. Current research indicates several possible mechanisms of interaction between SDF and BA. It may be due to the nonpolar surface of BA binding to the nonpolar methyl groups on the pectin chains [159]. Both BAs and pectin have hydrophobic groups, which may mediate adsorption through hydrophobic interactions [160]. The interaction between BAs and DF, but also the viscous chyme matrix formed by DF, can encapsulate bile salt micelles [161,162].

IDF has a lower BA-binding capacity and primarily plays an adsorptive role for BA [163]. Some studies have suggested that the interaction between DF and bile salts depends on the physicochemical properties of the fiber, including particle size and surface area, as well as the quantity and type of substituents, rather than the molecular weight of the fiber [164]. However, the adsorption capacity of lupin cotyledons, which is rich in both cellulose and pectin, for primary BAs is not related to the protein and DF content within them [160]. Therefore, the specific mechanisms of interaction between DF and BAs still need to be further elucidated.

### 5.2. Dietary Fiber Affects Bile Acid Metabolism Through the Microbiota in the Intestine

DF can influence the composition of gut microbiota, and certain microorganisms are involved in the conversion of secondary BAs. Therefore, DF can indirectly affect BA metabolism by altering the microbial composition. Table 2 presents examples of studies demonstrating how DF feeding in livestock leads to changes in specific microorganisms and BAs. Furthermore, studies in mice have shown that inulin fiber promoted microbiota-derived BAs and triggered inflammation [165]. Fructooligosaccharides have also been found to regulate the gut microbiome by enriching bacteria involved in the production of 6α-hydroxy BA, thereby activating the TGR5-GLP1R axis [157]. However, there is limited research on how DF influences BA metabolism through gut microbiota in livestock animals, and further studies are needed to clarify it.

## 6. Implications for Dietary Fiber Application Based on Microbial and Bile Acid Alterations

While studies on the DF–microbiota–BA axis improve our understanding of underlying biological mechanisms, such findings alone are not adequate to establish practical dietary fiber recommendations for animal nutrition without integrating performance and health-related outcomes.

In non-ruminant animals, mostly studied in pigs, supplementation with different types and levels of DF consistently induced alterations in gut microbial structure. Most studies indicated that low to moderate DF inclusion (approximately 0.05–15%) was associated with increased abundances of bacteria related to gut health, including *Lactobacillus*, *Bifidobacterium*, *Prevotella*, and *Faecalibacterium*. Meanwhile, reductions in *Helicobacter*, *Desulfovibrio*, *Proteus*, and some *Clostridium* taxa were also observed, suggesting a potential inhibitory effect on unfavorable microbial populations. Regarding BA metabolism, only a limited number of studies in pigs evaluated related parameters. These studies reported changes in BA composition, such as decreased CA and GDCA and increased UDCA and THCA, as well as shifts in BA proportions. However, current evidence mainly reflects alterations in BA profiles, and their physiological implications remain unclear.

In poultry, low-dose supplementation with functional fibers or oligosaccharides (e.g., FOS, MOS, and AXOS, approximately 0.05–0.5%) was commonly associated with increased abundances of *Lactobacillus* and *Bifidobacterium*, along with reductions in potential pathogens such as *Escherichia coli*, *Salmonella*, *Campylobacter*, and *Clostridium perfringens*. However, in a few high-fiber treatments (approximately 6–8% DDG and cereal by-products), increased abundances of *Escherichia coli* and *Campylobacter* were observed, indicating potential adverse effects at higher inclusion levels. Some studies also reported changes in BA composition, including alterations in CA, TCA, and DCA and reductions in LCA. These findings mainly reflect BA redistribution, and their functional relevance requires further investigation.

Studies in dogs showed that supplementation with fermentable or mixed DF (approximately 0.5–8%) was generally associated with increased abundances of *Bifidobacterium*, *Lactobacillus*, and *Faecalibacterium*, along with decreased *Fusobacterium*. Under some alternative formulations or high-protein/high-fiber conditions, reductions in *Bifidobacterium* or *Prevotella* were also reported. Regarding BA, some studies reported decreases in CA and DCA and increases in UDCA following DF or high-fiber diets, whereas high-dose prebiotic supplementation was occasionally associated with increased DCA and LCA, indicating dose-dependent effects. Most studies focused on DF inclusion levels between 1% and 8%. Higher levels (e.g., 25% grain-based diets) were mainly applied under specific experimental conditions, and their long-term safety remains uncertain. Data on cats remain limited.

In ruminants, DF is mainly supplied in the form of roughage or fibrous by-products. Moderate to relatively high DF supplementation (e.g., 10% alfalfa, 15–30% FDG, or high-NDF diets) was generally associated with increased abundances of fiber-degrading bacteria, including *Ruminococcus*, *Prevotella*, and *Butyrivibrio*, reflecting enhanced ruminal fiber fermentation. Some studies also reported decreases in *Clostridium*, *Blautia*, or *Bacteroides*, suggesting that changes in fiber structure and proportion may affect specific commensal populations. DF supplementation was associated with alterations in BA composition, including decreased CA, CDCA, and TCA and increased DCA and TDCA, mainly reflecting BA redistribution. However, direct functional evidence remains limited.

Based on the data collected in this review, most included studies mainly focused on changes in gut microbiota composition and BA profiles, whereas comprehensive health and production indicators, such as nutrient digestibility, growth performance, immune status, and metabolic responses, were not consistently evaluated. Therefore, in practical feeding and diet formulation, DF inclusion levels should be adjusted dynamically according to species, physiological stage, and fiber characteristics, as well as potential synergistic effects with other nutrients, and evaluated comprehensively using production performance and health status.

## 7. Conclusions

**Figure 1:**
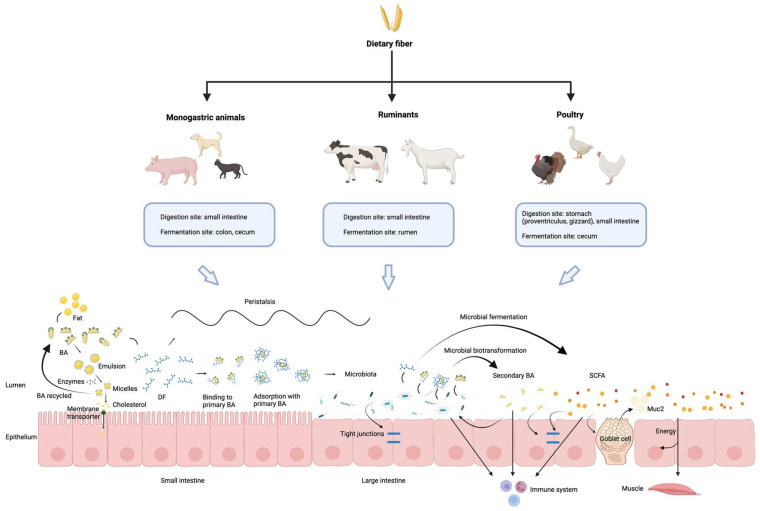
Overview of the main mechanisms underlying interactions between dietary fiber, microbiota and bile acids across different animal species. Dietary fiber digestion and fermentation vary among animal species. In the intestine, dietary fiber interacts with bile acids and gut microbiota, influencing lipid digestion, short-chain fatty acid production, intestinal barrier function, and immune regulation. Primary bile acids are partly reabsorbed in the ileum, while the remainder are transformed by gut microbiota into secondary bile acids in the colon, highlighting the dynamic fiber–microbiota–bile acid interactions along the gut. The schematic was created using BioRender.com (Created in BioRender. Lai, J. (2026) https://BioRender.com/ee7sdnh, accessed on 18 February 2026).

As summarized in Figure 1, this review comprehensively synthesizes the current understanding of DF in animal nutrition, focusing on its complex interplay with the gut microbiota and BA metabolism. DF is a fundamental component of animal diets, profoundly influencing gut health, overall metabolism, and immune function across various species. A significant insight from this review is the bidirectional relationship between BAs and the gut microbiota; the microbiota actively participates in BA transformation, and conversely, BAs influence the composition of the gut microbiota. Furthermore, DF not only impacts BA metabolism indirectly through microbial activity but also exhibits direct interactions with BAs. Despite extensive research, the precise and intricate mechanisms governing the crosstalk among DF, gut microbiota, and BA metabolism remain to be fully elucidated. Therefore, future research should aim to unravel these complex molecular and physiological pathways in greater detail.

## Figures and Tables

**Table 1:** Common dietary fiber sources and ingredients in animal diets.

Source (% DM)	SDF	IDF	ADF	NDF	ADL
Corn ^1^	0.90	6.00	2.90	9.10	0.30
Maize bran ^2^	3.20	24.00	14.50	44.20	2.20
Oat ^1^	3.60	9.80	13.70	25.30	-
Oat hull ^1^	4.90	65.70	32.10	65.90	5.40
Wheat bran ^1^	2.50	23.80	11.00	32.30	-
Soybean hull ^2^	1.96	22.39	46.20	64.40	2.30
Sugar beet pulp ^2^	29.00	20.70	24.80	49.50	1.80
Sunflower meal ^3^	1.48	14.62	25.40	37.45	6.48
Apple pomace ^3^	4.37	18.00	33.04	46.18	0.57
Corn-DDGS ^1^	3.00	14.10	12.00	30.50	2.60
DDGS ^3^	3.40	15.80	19.35	32.68	5.15
Barley ^1^	5.40	9.70	5.80	18.30	2.30
Rye ^1^	3.70	8.40	4.60	12.30	0.80
Sorghum ^1^	0.60	5.10	4.90	10.60	0.40
Sorghum hulls ^1^	10.00	45.00	41.60	59.40	-
Wheat ^1^	2.30	6.80	3.60	10.60	1.00
Pea hull ^2^	-	-	56.40	66.40	22.40
Rapeseed hull ^2^	-	-	42.20	55.80	22.70
Raw alfalfa meal ^4^	-	-	34.30	45.20	9.50
Raw amaranth grain ^5^	-	-	6.25	34.17	-
Rice bran ^6^	5.90	26.50	26.10	44.00	9.70

^1^ Do et al. [17]; ^2^ Hu et al. [18]; ^3^ Zhang et al. [19]; ^4^ Laudadio et al. [20]; ^5^ Kianfar et al. [21]; ^6^ Zhao et al. [22]. DM = dry matter; DDGS = distillers dried grains with solubles; SDF = soluble dietary fiber; IDF = insoluble dietary fiber; ADF = acid detergent fiber; NDF = neutral detergent fiber; ADL = acid detergent lignin.

**Table 2:** Influence of dietary fiber on the microbial composition and metabolism in different animals.

Animal Model (Breed/Strain), *n*	Physiological Stage/Age	Fiber Source and Level	Microbiota	Bile Acids	Reference
Pig					
Large × Landrace crossbred pigs, *n* = 7	Gestation	CON AM: 10% alfalfa meal BP: 10% beet pulp SH: 8% soybean skin	AM: ↑ *Paraprevotella*, Prevotellaceae NK3B31 group, *Lachnoclostridium* 1, *Eubacterium eligens* group, *Clostridium* sensu stricto 6, Lachnospiraceae NK4A136 group, *Clostridium* sensu stricto 1; ↓ *Helicobacter*, *Terrisporobacter*, *Desulfovibrio*, unclassified Lachnospiraceae, *Eubacterium fissicatena* group, Erysipelotrichaceae UCG 004, Ruminococcaceae V9D2013 group	Not reported	Liu et al. 2021 [38]
Duroc × Landrace × Large White crossbred piglets, *n* = 280	35 d	CG: control group soybean-meal diet, 2.27% CF AG: 5% alfalfa meal, 3.27% CF OG: 2% commodity concentrated fiber, 3.27% CF	AG: ↑ *Paenibacillus*, *Bacillus*, *Oceanobacillus*, *Lactococcus*, *Enterococcus*, *Exiguobacterium*; ↓ *Mycoplasma*, *Helicobacter* OG: ↑ *Paenibacillus*, *Faecalibacterium*; ↓ *Helicobacter*	Not reported	Liu et al. 2018 [39]
Large White pigs, *n* = 6	35 weeks	CON WW: whole wheat 10% AX: 10% wheat arabinoxylan 10% MLG: 10% oat-mixed linkage glucans AXMLG: 5% wheat arabinoxylan + 5% oat-mixed linkage glucans	10% MLG: ↑ *Prevotella*, *Mitsuokella*, *Lactobacillus*; ↓ *Clostridium*, *Mogibacterium*, *Streptococcus* AXMLG: ↑ *Prevotella*, *Mitsuokella*, *Lactobacillus*; ↓ *Clostridium*, *Mogibacterium*, *Streptococcus*	Not reported	Gorham et al. 2017 [22]
Growing pigs, *n* = 6	Growing	CON: fiber-free diet RS diet: 6.08% resistant starch β-glucan diet: 5.75% β-glucan xylan diet: 5.95% xylan	CON: ↑ *Lactobacillus* β-glucan diet: ↑ *Lactobacillus* Xylan diet: ↑ *Bifidobacterium*, *Blautia*, *Escherichia-Shigella*, Ruminococcaceae UCG-002, Ruminococcaceae UCG-005	Not reported	Wang et al. 2021 [40]
Duroc × (Landrace × Yorkshire) crossbred piglets, *n* = 36	28 d	CON CB: 5% corn bran WB: 5% wheat bran SB: 5% soybean hulls	CB or WB: ↑ Actinobacteria, Firmicutes, Fibrobacteres	Not reported	Zhao et al. 2018 [41]
Large White pigs, *n* = 25	Gestation	CON KON: 2.2% konjac flour	KON: ↑ Bacteroidota, Firmicutes, Spirochaetes, Verrucomicrobia; ↓ Actinobacteria, Proteobacteria	Not reported	Tan et al. 2016 [42]
German Landrace × Piétrain castrated pigs, *n* = 4	3 months	HF: high-fat/low-fiber (216.8 g NDF/kg DM) LF: low-fat/high-fiber (66.3 g NDF/kg DM)	LF: ↑ *Bifidobacterium* HF: ↑ *Bacteroides*, Enterobacteriaceae	Not reported	Heinritz et al. 2016 [43]
Yorkshire × Landrace pigs, *n* = 20	Gestation	CON LIG: 1.5% lignocellulose PRS: 2% resistant starch KON: 2% konjac flour	PRS: ↑ *Bacteroides*, *Parabacteroides*, *Turicibacter*; ↓ *Desulfovibrio*, *Oscillibacter* KON: ↑ *Bacteroides*, *Parabacteroides*; ↓ *Desulfovibrio*, *Oscillibacter*	Not reported	Lu et al. 2022 [44]
Yorkshire × Landrace pigs, *n* = 15	Gestation, lactation	CON: corn-soybean meal basal diet SBP: CON diet supplemented with 20% SBP in gestation and 10% SBP in lactation WB: CON diet supplemented with 30% WB in gestation and 15% WB in lactation	SBP: ↑ Christensenellaceae WB: ↑ Lactobacillaceae	Not reported	Shang et al. 2019 [45]
Duroc × Landrace piglets, *n* = 35	Weaned	CON: wheat-based control diet Treatment: CON supplemented with 15% wheat DDGS or 6% SBP inclusion	Treatment: ↑ *Lactobacillus*	Not reported	Thomson et al. 2012 [46]
Duroc × Landrace × Yorkshire piglets, *n* = 6	25 ± 1 d	CON: basal diet IDF: 1% IDF diet SDF: 1% SDF diet MDF: 0.5% IDF + 0.5% SDF diet	IDF: ↑ Bacteroidota, Euryarchaeota, *Phascolarctobacterium*, *Coprococcus*, *Prevotella* SDF: ↑ Proteobacteria, Actinobacteria, *Solobacterium*, *Succinivibrio*, *Blautia*, *Atopobium* MDF: ↑ Bacteroidota, Enterobacteriaceae, *Selenomonas*, *Phascolarctobacterium*, *Alloprevotella*	Not reported	Chen et al. 2019 [47]
Landrace × Yorkshire pigs, *n* = 6	Gestation, lactation	CON: basal diet Treatment: 20% alm kernel expeller	Treatment: ↑ *Lactobacillus*, Prevotellaceae, *Prevotella*; ↓ *Proteus*	Not reported	Ryu et al. 2022 [48]
Suhuai pigs, *n* = 7	Growing	0%, 7%, 14%, 21% or 28% defatted rice bran + basal diet	↑ unclassified Ruminococcaceae, Ruminococcaceae UCG*-010*, Lachnospiraceae, Erysipelotrichaceae UCG-004, *Acetitomaculum*, *Butyrivibrio*, *Akkermansia*; ↓ Rikenellaceae, unclassified Lachnospiraceae, *Campylobacter*, *Prevotella*, *Helicobacter*	Not reported	Pu et al. 2020 [49]
Large White pigs, *n* = 33/28	Gestation	CON 2% GCW: mixing 85.7% pregelatinized waxy maize starch with 14.3% guar gum	2% GCW: ↑ Unidentified Ruminococcaceae; ↓ CF231, L7A E11, *Clostridium*, *Lactobacillus*, *Bacteroides*, *Parabacteroides*, *Streptococcus*, *Dorea*, *Lachnospira*, *Bulleidia*	Plasma: ↓ total taurine-conjugated BAs, GUDCA, GCDCA, TUDCA, GLCA, TCDCA, CDCA-3Gln Fecal: ↑ HDCA, 3β-HDCA, MDCA, 7-KLCA, HCA, DCA, CDCA; ↓ DLCA	Wu et al. 2021 [50]
Duroc × Bamei crossbred pigs, *n* = 6	Not specified	0%, 10%, 17% or 24% broad bean silage + basal diet	↑ *Prevotella*, *Bacteroides*, *Ruminococcus*, *Oscillibacter*, *Parabacteroides*; ↓ unclassified Enterobacteriaceae, *Streptococcus*	↓ CA, CDCA, TCDCA, GCDCA, TCA, GCA	Tang et al. 2024 [51]
Duroc × Landrace × Yorkshire crossbred pigs, *n* = 8	33 ± 1 d	NS: fiber-free diet SI: fiber-free diet + 3% FOS MIX: fiber-free diet + 3% DF mixture (FOS, long-chain inulin, and microcrystalline cellulose at the ratio 1:1:1)	SI: ↑ gut bacteria capable of expressing 7α-hydroxysteroid dehydrogenase (7α-HSDH)	SI: ↑ THCA, α-MCA; ↓ GDCA MIX: ↑ THCA, CA; ↓ GDCA	Hu et al. 2023 [52]
Duroc × Landrace × Yorkshire crossbred pigs, *n* = 60	28 d	CON XOS: 0.05% xylooligosaccharide	XOS: ↑ unclassified *Lactobacillus*, *Lactobacillus johnsonii*, *Lactobacillus amylovorus*, unclassified *Lactobacillus*; ↓ *Clostridium* sensu stricto 1, unclassified Peptostreptococcaceae, *Intestinibacter*	XOS: ↑ UDCA; ↓ CA	Tang et al. 2022 [53]
Poultry					
Cobb 500 broiler chickens, *n* = 160	28/42 d	CTL^−^: antibiotic-free CTL^+^: 11 mg/kg of virginiamycin MOS: 0.2% mannanoligosaccharides to 21 d and 0.1% mannanoligosaccharides thereafter LL: 1.25% purified lignin HL: 2.5% purified lignin	MOS: ↑ *Lactobacillus*, *Bifidobacterium*; ↓ *Escherichia coli* LL: ↑ *Lactobacillus*; ↓ *Escherichia coli* HL: ↑ *Lactobacillus*; ↓ *Escherichia coli*	Not reported	Baurhoo et al. 2007 [54]
Kabir strain broiler chickens, *n* = 14	40 d	0.5% FOS + 3% GOS	↑ *Bifidobacterium*; ↓ *Campylobacter*	Not reported	Baffoni et al. 2012 [55]
Broiler chickens, *n* = 2500	7–35 d	CON MOS: 0.08% MOS	MOS: ↑ *Lactobacillus*, *Bifidobacterium*; ↓ *Salmonella*, *Escherichia coli*, *Clostridium perfringens*, *Campylobacter*	Not reported	Corrigan et al. 2015 [56]
Ross broiler chickens, *n* = 40	28 d	CON Avilamycin (6 mg/kg) 0.25% FOS 0.5% FOS 0.025% MOS 0.05% MOS	0.25% FOS: ↑ total bacteria, *Lactobacillus*; ↓ *Clostridium perfringens*, *Escherichia coli* 0.05% MOS: ↑ total bacteria, *Lactobacillus*; ↓ *Clostridium perfringens*, *Escherichia coli*	Not reported	Kim et al. 2011 [57]
Broiler chickens, *n* = 56	21 d	Ara-binose-to-xylose ratio of 0.25 for treatment group CON 0.2% AXOS-3-0.25 arabinoxylooligosaccharides 0.2% AXOS-9-0.25 0.4% AXOS-9-0.25	0.4% AXOS-9-0.25: ↓ *Salmonella*	Not reported	Eeckhaut et al. 2008 [58]
Ross 308 broilers and Hy-line W36 laying-hen chickens, *n* = 88	21 d	LF (low fiber): 1–21 d low fiber (basic diet) HF (high fiber): 1–12 d 6% DDG and 6% wheat/12–21 d 8% DDG and 8% wheat	broilers/HF: ↑ Selenomonadales, Enterobacteriales, Campylobacterales, *Escherichia coli*, *Campylobacter* genus laying-hen chicks/HF: ↑ *Escherichia coli* laying-hen chicks or broilers/LF: ↓ *Bacteroides* genus	Not reported	Walugembe et al. 2015 [59]
Ross 308 broiler chickens, *n* = 49	42 d	0%, 0.2%, 0.4%, 0.6% or 0.8% lactulose	↑ *Lactobacillus*	Not reported	Calik et al. 2015 [60]
Arbor Acresbroiler chickens, *n* = 40	21 d	NF: standard maize–soybean meal diet CON: DF deprivation diet Glu: Con + 3% β-glucan Ara: Con + 3% arabinoxylan RS: Con + 3% resistant starch	NF: ↓ *Butyricicoccus* Glu: ↑ *Lactobacillus* RS: ↑ *Bacteroides* Ara: ↑ *Coprococcus*	Plasma Ara: ↑ CA, TCA, GCA, DCA, TDCA, TLCA; ↓ LCA Liver Glu: ↓ LCA RS: ↓ LCA Ara: ↑ TCA, GCA, TDCA, TLCA; ↓ LCA	Yang et al. 2024 [61]
Quails, *n* = 21	35 d	0, 5, 10 and 15 g/kg micronised wheat fiber	No significant difference	Not reported	Rezaei et al. 2018 [62]
Dog					
Dogs, *n* = 3	Not specified	Diet 1: low-protein diet (crude protein (CP) 229 g/kg dry matter (DM)) Diet 2: high-protein diet (CP 304 g/kg DM) Diet 3: Diet 1 + 1.5 g of FOS/kg Diet 4: Diet 2 + 1.5 g of FOS/kg	No significant difference	Not reported	Pinna et al. 2018 [63]
Beagle dogs, *n* = 4/5	8.7 ± 2.0 years	3% chicory (1.5% inulin)	↑ *Bifidobacterium*	Not reported	Zentek et al. 2003 [64]
Dogs, *n* = 6	20 months	7.5% SBP	↑ Firmicutes; ↓ *Fusobacterium*	Not reported	Middelbos et al. 2010 [65]
Shiba dogs, *n* = 1/3	7–48 months	5.6% soybean husk power or cellulose powder	↑ *Lactobacillus*, *Faecalibacterium prausnitzii*, *Clostridium* cluster XIVa, *Bacteroides-Prevotella-Porphyromonas* group; ↓ *Clostridium* cluster XI	Not reported	Myint et al. 2017 [66]
Beagle dogs, *n* = 8	15 months (7 dogs) or 6 years (1 dog)	High SBP: 12% SBP (3.1% crude fiber) Low SBP: 2.7% SBP (0.96% crude fiber) LC: 2.7% lignocellulose (2.4% crude fiber)	Low SBP or LC vs. high SBP: ↓ *Clostridium coccoides* cluster, *Clostridium* leptum cluster, *Lactobacillus*, *Bifidobacterium*	Not reported	Kröger et al. 2017 [67]
Dogs, *n* = 6	4.5 years	CON: no supplemental fiber 2.5% BP 2.5% cellulose CF: 1% cellulose + 1.5% FOS CFY1: 1% cellulose + 1.2% FOS + 0.3% yeast cell wall CFY2: 1% cellulose + 0.9% FOS + 0.6% yeast cell wall	Beet pulp, CF, CF1 and CF2 (diets containing fermentable fiber): ↑ *Lactobacillus*, *Bifidobacterium*	Not reported	Middelbos et al. 2007 [68]
Beagle dogs, *n* = 12	3–4 years	Equivalent insoluble/soluble ratio CTR: low-fiber diet BRA: 10% wheat midds + 6% oat bran + 1.5% oat fiber + 5.5% SBP FRU: 5% citrus pulp + 3.5% apple fiber + 0.6% orange peel + 0.5% pomegranate peel + 2.5% cellulose pellets	BRA: ↑ *Lachnospira*, *Bifidobacterium*, *Faecalibacterium*	Not reported	Montserrat-Malagarriga et al. 2024 [69]
Dogs, *n* = 18	5.7 ± 2.6 years	RYE: 25% whole-grain rye OAT: 25% oats WHE: 25% wheat	RYE: ↑ *Prevotella* 9; ↓ Lachnospiraceae, *Bacteroides* RYE vs. WHE or OAT: ↓ *Bacteroides* RYE vs. WHE: ↑ *Catenibacterium* RYE vs. OAT: ↑ *Megamonas*	Not reported	Palmqvist et al. 2023 [70]
Dogs, *n* = 6	4.6 ± 0.95 years	W: 269 g/kg wheat RW: mixed 132 g/kg rye/135 g/kg wheat R: 264 g/kg rye	R: ↑ *Prevotella*; ↓ *Romboutsia*, unclassified member of the family Peptostreptococcaceae	Not reported	Palmqvist et al. 2023 [71]
Beagle dogs, *n* = 6	5.5 ± 0.5 years	Beef control Beef + 1.4% inulin Beef + 1.4% yeast cell wall extract (YCW) Chicken control Chicken + 1.4% inulin Chicken + 1.4% YCW	1.4% inulin: ↑ *Megamonas*, *Lactobacillus*; ↓ Enterobacteriaceae 1.4% yeast cell wall extract: ↑ *Bifidobacterium* 1.4% inulin vs. 1.4% yeast cell wall extract: ↑ *Lactobacillus*; ↓ *Escherichia coli*	Not reported	Beloshapka et al. 2013 [72]
Dogs, *n* = 10	6.13 ± 0.17 years	0%, 1.5%, 3%, 4.5% or 6% potato fiber	↑ Firmicutes, *Faecalibacterium*; ↓ *Fusobacterium*	Not reported	Panasevich et al. 2015 [73]
Beagle dogs, *n* = 8	3.02 ± 0.71 years	COSP: CON containing moderate protein and fiber HPHF: high-protein, high-fiber HPHFO: high-protein, high-fiber plus omega-3 and medium-chain fatty acids	HPHF and HPHFO: ↑ *Faecalibacterium*, *Romboutsia*, *Fusobacterium*; ↓ *Catenibacterium*, *Bifidobacterium*, *Prevotella 9*, *Eubacterium*, *Megamonas*	HPHFO: ↓ CA, total primary BA	Phungviwatnikul et al. 2021 [74]
Dogs, *n* = 16–17	Beagles: 24.2 ± 7.1 months; mixed-breed hounds: 13.3 ± 0.8 months	HA-GI: high animal protein, grain-inclusive LA-GF: low animal protein, grain-free LA-GI: low animal protein, grain-inclusive HA-GF: high animal protein, grain-free	LA-GF vs. HA-GI/LA-GI/HA-GF: ↑ Selenomonadaceae, Veillonellaceae, Lactobacillaceae, *Streptococcus*, *Ligilactobacillus*, *Megamonas*, *Collinsella aerofaciens*, *Bifidobacterium*	LA-GF vs. HA-GI/LA-GI/HA-GF: ↓ CA LA-GF vs. HA-GI/HA-GF: ↓ DCA	Clark et al. 2023 [75]
Beagle dogs, *n* = 12	5.5 ± 1.1 years	HPHF: high-protein, high-fiber (TDF: 26.81% DM, barley, BP, cellulose, psyllium husk, scFOS, and brown flax seed)	HPHF: ↑ Proteobacteria, Coriobacteriaceae UCG-002, undefined Muribaculaceae, *Ruminococcus gauvreauii* group, uncultured Erysipelotrichaceae, *Bifidobacterium*, *Allobaculum*, *Eubacterium*, *Negativibacillus*, *Parasutterella*; ↓ Prevotellaceae Ga6A1 group, *Ruminococcus gnavus* group, *Catenibacterium*, *Erysipelatoclostridium*, *Holdemanella*, *Lachnoclostridium*, *Lactobacillus*, *Megamonas*, *Peptoclostridium*, *Streptococcus*	HPHF: ↑ UDCA; ↓ DCA	Phungviwatnikul et al. 2022 [76]
Beagle dogs, *n* = 3	4.16 years	Control: non-prebiotic, Low: 0.5% inulin-type fructans High: 1% inulin-type fructans	Low: ↑ *Eubacterium* High: ↓ *Coprobacillus*	Low: ↓ DCA, LCA, TBA High: ↑ DCA, LCA, TBA	Alexander et al. 2018 [77]
Cat					
Cats, *n* = 4	1.7 ± 0.1 years	4% cellulose, 4% FOS or 4% pectin	FOS: ↑ *Bifidobacterium*; ↓ *Escherichia coli* Pectin: ↑ *Clostridium perfringens*, *Escherichia coli*, *Lactobacillus*	Not reported	Barry et al. 2010 [33]
Cats, *n* = 2	2.8 years	no prebiotic 0.5% scFOS 0.5% GOS 0.5% scFOS + 0.5% GOS	0.5% scFOS + 0.5% GOS: ↑ *Bifidobacterium*	Not reported	Kanakupt et al. 2011 [78]
Cats, *n* = 12	1–10 years	0.45% FOS + inulin	↑ Veillonellaceae; ↓ Gammaproteobacteria	Not reported	Garcia-Mazcorro et al. 2017 [79]
Cats, *n* = 8	6.4 years	CON 2% wool hydrolysate 2% inulin 2% cellulose	2% wool hydrolysate: No changes 2% inulin: ↑ *Catenibacterium*, *Bulleidia*, *Bifidobacterium*; ↓ *Fusobacterium*, *Faecalibacterium*, *Coprococcus*, *Allobaculum, Slackia*	Not reported	Deb-Choudhury et al. 2018 [80]
Cattle/Bovidae					
Holstein dairy bull calves, *n* = 6	7 d	MS: milk and starter for the control group MSO2: supplementation of 10% oat hay from week 2 on the basis of milk and starter MSO6: supplementation of 10% oat hay from week 6 on the basis of milk and starter	MSO2 and MSO6 vs. MS: ↓ *Butyricimonas*, *Parabacteroides*, *Porphyromonas*, *Anaerotruncus*, *Blastopirellula, Comamonas* MSO6 vs. MSO2 and MS: ↓ *Desulfovibrio*	Not reported	Lin et al. 2018 [81]
Yak calves, *n* = 5	Pre-weaning	CON: milk replacer A: milk replacer with 10% alfalfa hay S: milk replacer with starter feed SA: milk replacer with starter feed plus 10% alfalfa hay	SA or A vs. S or CON: ↑ *Desulfobulbus*, *Olsenella*, *Pseudoflavonifractor*, *Stomatobaculum*; ↓ *Blautia*, *Clostridium* IV, *Bacteroides*, *Eubacterium*, *Clostridium*	Not reported	Wu et al. 2021 [82]
Yak calves, *n* = 5	30 d	CON A: alfalfa hay S: starter feeding SA: starter plus alfalfa hay	SA: ↑ *Limnobacter*, *Escherichia/Shigella*, *Aquabacterium*, *Coprococcus*, *Pseudobutyrivibrio*, *Flavonifractor*, *Synergistes, Sutterella*	Not reported	Cui et al. 2020 [83]
Guanling cattle, *n* = 6	18 months	BD: the treatments included a basal diet 15% FDG: a 15% concentrate replaced by FDG in the basal diet 30% FDG: a 30% concentrate replaced by FDG in the basal diet	15% FDG: ↑ Ruminococcaceae UCG-010; ↓ *Treponema* 2 30% FDG: ↑ Ruminococcaceae UCG-010; ↓ *Treponema* 2	15% FDG: ↓ CDCA, CA, TCA 30% FDG: ↓ CDCA, CA, TCA	He et al. 2023 [84]
Dairy cows, *n* = 6	Day in milk, 233 ± 23 d; parity, 2	CON: NFC/NDF = 0.97 HG (high-grain): NFC/NDF = 1.42	↑ *Paraclostridium*, *Anaerobutyricum*, *Shuttleworthia*, *Stomatobaculum*	HG: ↑ 7-DHCA; ↓ TCA, GCA, TDCA, TCDCA, TLCA	Lai et al. 2023 [85]
Dairy cows, *n* = 8	Day in milk, 134 ± 7.0 d; parity, 3.12 ± 0.610	0, 100, 200, 300 and 400 inulin g/d	↑ *Bacteroides*, *Bifidobacterium*; ↓ Ruminococcaceae, clostridia, *Paeniclostridium*, *Coprococcus*	Feces ↑ DCA, TDCA, CCA Serum ↑ GCA, TDCA, TCA, THCA	Wang et al. 2022 [86]
Sheep					
Ovis aries, “Assaf” breed lambs, *n* = 19/15	After birth	CON 11.4% FOS and garlic residues	↑ *Bifidobacterium*, *Enterococcus*, *Lactobacillus*, *Veillonella*	Not reported	Quijada et al. 2020 [87]
Dorper × small-tailed Han hybrid sheep, *n* = 6	2 months	Soluble fraction and insoluble fraction of NDF, WPCS (19.7% SF and 37.58 IF), WPCSB (22.2% SF and 32.45% IF) WPCS: untreated whole-plant corn silage WPCSB: WPCS inoculated with bacterial inoculant	WPCSB vs. WPCS: ↑ Bacteroidota, *Prevotella*; ↓ Firmicutes, *Bacteroides*, *Selenomonas*, *Clostridium*, *Ruminococcus*	Not reported	Guo et al. 2022 [88]
Hu sheep, *n* = 4	3 months	Forage: concentrate ratio of 60:40 on a dry matter basis AH: 40% alfalfa hay RS: rice straw BF: bio-fermented rice straw	AH: ↑ *Ruminococcus* RS: ↑ Bacteroidales UCG 001 BF: *Prevotella*, unclassified Muribaculaceae	BF vs. RS: ↑ TCA, TCDCA	Kyawt et al. 2024 [89]
Gansu alpine fine wool ewes, *n* = 40	3 to 4 years	Alfalfa supplementary H: NFC/NDF = 1.92 M: NFC/NDF = 1.11 L: NFC/NDF = 0.68	H group: ↑ Ruminococcaceae, *Ruminococcus*; ↓ *Prevotella*	Not reported	Chen et al. 2024 [90]
Goat					
Arbas goats, *n* = 8	6 months	OAT: 35% whole oat OA73: 24.5% oat + 10.5% alfalfa OA37: 10.5% oat + 24.5% alfalfa Alfalfa: 35% whole alfalfa	Alfalfa vs. OAT: ↑ Rikenellaceae RC9 gut group, unclassified Bacteroidales; ↓ *Clostridium*	Not reported	Sun et al. 2022 [91]

CON = control; IDF = insoluble dietary fiber; SDF = soluble dietary fiber; SBP = sugar beet pulp; KON = konjac flour; DDGS = distillers dried grains with solubles; XOS = xylooligosaccharides; FOS = fructooligosaccharides; MOS = mannanoligosaccharides; GOS = galactooligosaccharides; LIG = lignocellulose; CF = crude fiber; RS = resistant starch; WB = wheat bran; CB = corn bran; SB = soybean hulls; CA = cholic acid; CCA = cholanic acid; CDCA = chenodeoxycholic acid; DCA = deoxycholic acid; DLCA = dehydrolithocholic acid; GCA = glycocholic acid; GCDA = glycodeoxycholic acid; GCDCA = glycochenodeoxycholic acid; GLCA = glycolithocholic acid; GUDCA = glycoursodeoxycholic acid; HCA = hyocholic acid; HDCA = hyodeoxycholic acid; LCA = lithocholic acid; MDCA = murideoxycholic acid; TCA = taurocholic acid; TCDCA = taurochenodeoxycholic acid; TDCA = taurodeoxycholic acid; THCA = taurohyodeoxycholic acid; TLCA = taurolithocholic acid; TUDCA = tauroursodeoxycholic acid; TBA = total bile acids; UDCA = ursodeoxycholic acid; 7-KLCA = 7-ketolithocholic acid. ↑ indicates an increase, and ↓ indicates a decrease in the abundance of gut microbiota or in bile acid levels. Not all studies included bile acid measurements, and bile acid profiles are not available for all entries.

**Table 3:** Effects of microorganisms on bile acid metabolism in different animals.

Animal	Microbial Intervention	Effect on BA Metabolism	Reference
Pig	Fecal microbiota transplantation	Altered BA cycling in the small intestine	Teng et al. 2023 [124]
Pig	*Lactobacillus delbrueckii*	↑ Fecal BA excretion and hepatic enzyme activity related to BA synthesis	Hou et al. 2024 [125]
Pig	*Bacillus subtilis* fermented liquid	↑ BSH- and 7α-dehydroxylase-active bacteria; ↓ unconjugated BA production	He et al. 2017 [126]
Poultry	*Clostridium butyricum*	↑ TUDCA, LCA; ↓ TαMCA	Wang et al. 2020 [127]
Poultry	*Candida tropicalis* ZD-3	↑ NorCA, NorDCA, TLCA; ↓ 3β-CA	Feng et al. 2024 [128]
Poultry	*Lactobacillus plantarum* P8	↑ DCA	Zhao et al. 2023 [129]
Dogs/cats	*Peptacetobacter* (*Clostridium*) *hiranonis*; Oscillospirales	Conversion of primary BAs to secondary BAs via *bai* operon and BSH activity	Correa Lopes et al. 2024; Rowe et al. 2024 [130,131]
Ruminants	Rumen microorganisms (BSH, baiN, BASS-related)	Involved in BA metabolism through enzymatic activities	Asar et al. 2024; Zhang et al. 2024 [132,133]
Ruminants	*Saccharomyces cerevisiae* supplementation	↑ Secondary BA-related microbes in feces	Li et al. 2023 [134]

BA = bile acid; BSH = bile salt hydrolase; TUDCA = tauroursodeoxycholic acid; LCA = lithocholic acid; DCA = deoxycholic acid; NorCA = norcholic acid; NorDCA = nordeoxycholic acid; TαMCA = tauro-α-muricholic acid; BSH = bile salt hydrolase; baiN = bile acid-inducible gene N; BASS-related = bile acid sodium symporter-related protein. ↑ indicates an increase and ↓ indicates a decrease in the levels, abundance, or activity of bile acids, related microbes, and metabolic processes.

**Table 4:** Effects of bile acids on gut microbiota in different animals.

Animal	BA Intervention	Effect on Microorganism	Reference
Pig	Oleanolic acid (BA receptor agonist)	Suppressed *Bacteroides* expansion and mitigated the reduction in microbial diversity induced by parenteral nutrition	Jain et al. 2017 [141]
Pig	CDCA supplementation	↑ *Prevotella 9*, Prevotellaceae *TCG-001*; ↓ *Dorea*	Song et al. 2021 [142]
Pig	Porcine BA extracts	No significant changes in fecal microbiota; ↑ secondary BA-related metabolites	Zhou et al. 2023 [143]
Poultry	Bile, TCDCA, or TCA	*Turicibacter bilis* MMM721 cultures: altered expression of proteins related to ribosomal processes, chaperones, and cell surface modification	Maki et al. 2022 [144]
Laying hens	90 mg/kg BA supplementation	↑ *Lactobacillus*, *Bifidobacterium*, *Turicibacter*	Yang et al. 2022 [145]
Broilers	DCA supplementation	↑ Bacteroidota; ↓ Firmicutes, *Clostridium perfringens*, *Campylobacter jejuni*	Bansal et al. 2020; Bansal et al. 2021; Alrubaye et al. 2019 [146,147,148]
Broilers	BA (HCA, HDCA, CDCA) supplementation	↑ *Akkermansia*	Wang et al. 2024 [149]
Broilers	BA supplementation	↑ *Bifidobacterium*, *Escherichia coli*, *Lactobacillus*; ↓ *Bacteroides*	Wang et al. 2024 [149]
Broilers	BA supplementation	↑ *Lactobacillus*, *Anaerostipes*, *Sellimonas*, CHKCI002; ↓ *Barnesiella*, *Akkermansia*	Hu et al. 2024 [150]
Broilers	BA supplementation	↑ Bacteroidota, *Bacteroides*; ↓ Campylobacteraceae, *Campylobacter*	Hu et al. 2024 [151]
Goats	BA supplementation	↑ *Akkermansia*; ↓ *Prevotella*, *Treponema*	Hou et al. 2024 [152]
Dairy calves	UDCA derived from gut microbiota	↓ ESBL-producing *Escherichia coli*	Singh et al. 2019 [100]
Dairy goats	BA supplementation	↑ *Candidatus Saccharimonas*, *Eubacterium coprostanoligenes* group, *Akkermansia*, *Subdoligranulum*	Yin et al. 2024 [153]

BA = bile acid; CDCA = chenodeoxycholic acid; DCA = deoxycholic acid; TCA = taurocholic acid; TCDCA = taurochenodeoxycholic acid; UDCA = ursodeoxycholic acid; HCA = hyocholic acid; HDCA = hyodeoxycholic acid; ESBL = extended-spectrum beta-lactamase. ↑ indicates an increase and ↓ indicates a decrease in the abundance, activity, or related metabolic features of gut microbiota.

## Data Availability

No new data were created or analyzed in this study.

## Abbreviations

The following abbreviations are used in this manuscript:

AACC	American Association of Cereal Chemists
AC	Adenylate cyclase
ADF	Acid detergent fiber
ADL	Acid detergent lignin
ASBT	Apical sodium-dependent BA transporter
BAs	Bile acids
BAAT	Amino acid N-acyltransferase
BACS	BA-CoA synthetase
baiN	3-dehydro-BA delta4,6-reductase
BASS	BA: Na^+^ symporter family
BSEP	Bile salt export pump
BSH	Bile salt hydrolase
CA	Cholic acid
cAMP	Cyclic adenosine monophosphate
CCA	Cholanic acid
CCK	Cholecystokinin
CDCA	Chenodeoxycholic acid
CRC	Colorectal cancer
CYP27A1	27-hydroxylase
CYP7A1	7α-hydroxylase
CYP7B1	7α-hydroxylase
CYP8B1	12α-hydroxylase
CYPs	Cytochrome P450
DF	Dietary fiber
DCA	Deoxycholic acid
DLCA	Dehydrolithocholic acid
DDGS	Distillers dried grains with solubles
DM	Dry matter
ESBL	Extended-spectrum beta-lactamases
FGF15/19	Fibroblast growth factor 15/19
FGFR4	Fibroblast growth factor receptor 4
FXR	Farnesoid X receptor
GCA	Glycocholic acid
GCDCA	Glycochenodeoxycholic acid
GDCA	Glycodeoxycholic acid
GLCA	Glycolithocholic acid
GLP-1/2	Glucagon-like peptides-1/2
GPCR	G protein-coupled receptor
GUDCA	Glycoursodeoxycholic acid
HCA	Hyocholic acid
HDCA	Hyodeoxycholic acid
HSDHs	Hydroxy steroid dehydrogenases
IDF	Insoluble dietary fiber
IL-1β	Interleukin-1 beta
ILC3	Innate lymphoid cells type 3
KO	Knockout
LCA	Lithocholic acid
LRH-1	Liver receptor homolog-1
MDCA	Murideoxycholic acid
MCP-1	Monocyte chemoattractant protein-1
MRP2	Multidrug resistance-associated protein 2
NDF	Neutral detergent fiber
NorCA	Norcholic acid
NTCP	Sodium/taurocholate co-transporting polypeptide
OATP	Organic anion-transporting polypeptides
OSTα/OSTβ	Organic solute transporters alpha and beta
PXR	Pregnane X receptor
RORγt	Retinoic acid-related orphan receptor gamma t
SARA	Subacute ruminal acidosis
SCFAs	Short-chain fatty acids
SDF	Soluble dietary fiber
SHP	Small heterodimer partner
SLC	Sulfolithocholate
TBA	Total bile acids
TCA	Taurocholic acid
TCDCA	Taurochenodeoxycholic acid
TDCA	Taurodeoxycholic acid
TGR5	Takeda G-protein-coupled receptor 5
THCA	Taurohyodeoxycholic acid
TLCA	Taurolithocholic acid
TNF-α	Tumor necrosis factor-alpha
Tregs	Regulatory T cells
TUDCA	Tauroursodeoxycholic acid
TαMCA	Tauro-α-muricholic acid
UDCA	Ursodeoxycholic acid
VDR	Vitamin D receptor
7-KLCA	7-ketolithocholic acid
